# Influence of Shelf-Aging on Resistive Switching Characteristics of Sol–Gel-Processed ZnO and Al-Doped ZnO Structures

**DOI:** 10.3390/nano16191263

**Published:** 2026-10-07

**Authors:** Dongjin Moon, Jaehoon Kim

**Affiliations:** Department of Semiconductor Engineering, Gachon University, Seongnam-si 13120, Gyeonggi-do, Republic of Korea

**Keywords:** resistive switching devices, memristors, quantum dot light-emitting diodes, ZnO, Al-doped ZnO, positive aging, shelf-aging

## Abstract

Sol–gel-processed zinc oxide (ZnO) is indispensable in various optoelectronic applications, such as quantum dot light-emitting diodes (QLEDs), due to its high electron transport capability and adjustable oxygen vacancy characteristics. However, its commercialization has been delayed by defect-related issues and a high sensitivity to fabrication conditions. ZnO exhibits complex aging behaviors, notably ‘shelf-aging,’ where device properties change unpredictably over time even without external stimuli. Recent studies suggest that this shelf-aging in QLEDs might relate to the resistive switching (RS) behavior in ZnO, affecting the current conduction mechanism through external bias and internal oxygen dynamics. Despite its significance, systematic studies on the shelf-aging effects in ZnO-based RS structures have been lacking. In this study, we fabricate RS devices using sol–gel-processed ZnO and aluminum-doped ZnO (AZO) as dielectric layers, employing indium tin oxide (ITO) and aluminum (Al) as electrodes—materials commonly utilized in QLEDs. Notably, the *I*_on_/*I*_off_ ratio increased during the initial storage period, reaching the highest measured values after two days for ZnO and three days for AZO, followed by a decrease at later storage times. Further electrical measurements revealed storage-associated changes, while qualitative time-of-flight secondary ion mass spectrometry (TOF-SIMS) profiles showed differences between pristine and archived aged specimens. These observations suggest possible interfacial or defect-related contributions. Overall, this study documents electrical evolution in simplified oxide/Al-containing RS structures during ambient storage with repeated measurements and generates hypotheses for future investigation into complete QLEDs.

## 1. Introduction

**Figure 1:**
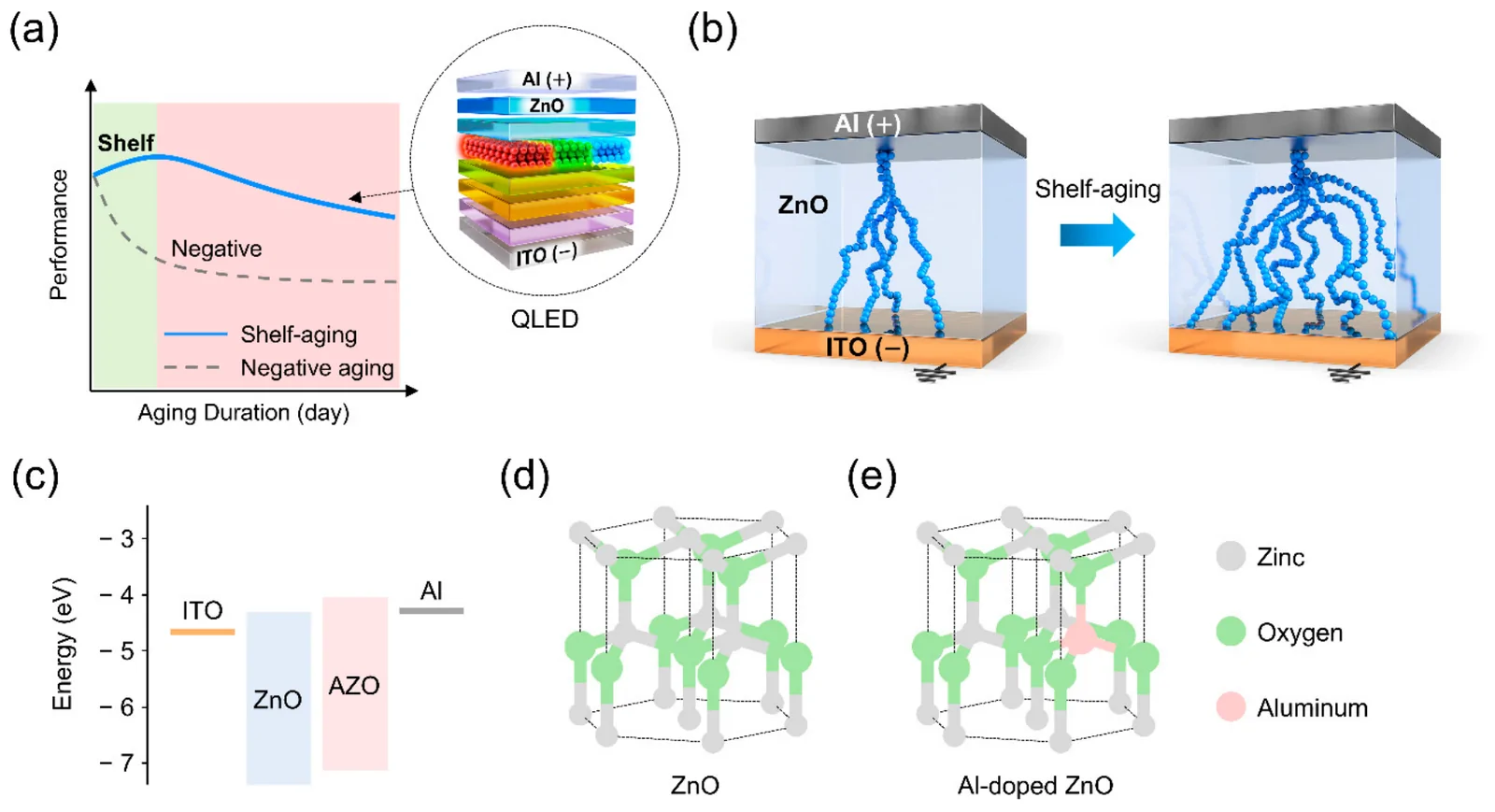
Schematic illustrations of (**a**) performance variation in shelf-aged QLEDs, (**b**) CF variation in a shelf-aged sol–gel-processed RS device, (**c**) energy band diagrams, and (**d**,**e**) schematic structural models of ZnO and AZO.

In next-generation optoelectronics [1], thin-film [2], and memory devices [3,4], zinc oxide (ZnO) and metal-doped ZnO are widely utilized. Specifically, in optoelectronics, ZnO is nearly indispensable as an electron transport layer (ETL) in quantum dot light-emitting diodes (QLEDs) [5], where it facilitates electron injection into the quantum dot (QD) emissive layer (EML). Although research has explored replacing ZnO with organic ETLs due to its highly sensitive surface nature and the significant variability in device characteristics depending on synthesis conditions [6,7], the performance of QLEDs based on ZnO has yet to be matched in terms of efficiency and lifetime. However, ZnO has not yet been actively commercialized as an ETL for self-emitting display technology for several reasons, one of the most important being its complex aging characteristics [8]. In display panel manufacturing, predictable behavior based on biexponential aging characteristics is essential for designing compensation circuits that adjust brightness, ensuring panels maintain consistent luminance [9]. However, QLEDs utilizing ZnO and various metal-doped ZnOs as the ETL do not age uniformly; they exhibit either an initial increase in efficiency (positive aging) or arbitrary aging characteristics, where brightness decreases in an inconsistent manner [10]. This phenomenon, known as ‘positive aging,’ is influenced by various factors. For instance, a study first reported in 2017 suggested that acidic components—most notably acrylic acid—in ultraviolet-curable resins used for encapsulation react with ZnO to form zinc carbonate at the ZnO surface, which acts as a passivation layer [11]. Other aging factors, such as heat and light, have also been identified [12,13]. In particular, ‘shelf-aging’—where device performance varies even without external stimuli—is known to influence the positive aging of QLEDs (Figure 1a,b). For example, a previous report in 2018 indicated that during shelf-aging, reactions at the interface between Mg-doped ZnO (ZnMgO) and aluminum (Al) lead to the formation of aluminum oxide (AlO*_x_*), which not only passivates defect states on the ZnMgO surface but also increases oxygen vacancies within ZnMgO, enhancing conductivity [14]. This increase in conductivity boosts electron injection and optimizes the electron–hole balance.

Furthermore, recent studies have begun to reveal that the resistive switching (RS) characteristics of ZnO or metal-doped ZnO in QLEDs influence the arbitrary aging of these devices. RS refers to a change in the electrical resistance of a material, typically shifting between a high-resistance state (HRS) and a low-resistance state (LRS), controlled by an applied voltage. According to a recent study, during QLED operation, the formation of a conductive filament (CF) causes the electrical state of the ZnO layer to transition from HRS to LRS, significantly increasing electron injection and thereby improving the electron–hole balance [15]. Furthermore, while their study provided valuable insights into the possible correlation between QLED positive aging and RS, it did not include a systematic analysis, particularly regarding shelf-aging, which has the most significant impact on the positive aging of QLEDs. Additionally, an investigation into how RS characteristics evolve with aging was not the primary focus, as their work was centered on identifying the presence of RS characteristics in ZnMgO-based RS devices. Thus, the oxide/Al interface provides a motivation for studying simplified RS structures alongside the QLED literature.

Therefore, the present work examines the evolution of RS characteristics in ITO/ZnO/Al and ITO/Al-doped ZnO (AZO)/Al structures during ambient storage with intermittent electrical measurements. These simplified structures serve as a proxy platform for generating hypotheses concerning oxide/Al-related electrical changes that may warrant future investigation in complete QLEDs. These devices are fabricated using methods such as sol–gel processing, rather than sputtering, which is commonly employed in memory studies [16]. AZO is specifically evaluated alongside ZnO, as will be discussed later, because Al plays a crucial role in shelf-aging. By intentionally doping ZnO with Al, this study seeks to gain deeper insights into the role of Al in the aging process. These devices utilize indium tin oxide (ITO) and Al as the bottom and top electrodes, respectively, both of which are commonly used in QLED devices [10,11,13,14]. The results of this study indicate that both ITO/ZnO/Al and ITO/AZO/Al RS devices exhibit a significant increase in the *I*_on_/*I*_off_ ratio following shelf-aging. Notably, in ZnO, the current scale decreases by more than two orders of magnitude with shelf-aging, while in AZO, the current scale initially increases by more than two orders of magnitude. Within the sampled storage times, the highest measured *I*_on_/*I*_off_ ratios were observed after 2 days for ZnO and 3 days for AZO, followed by lower values at the later measured storage times. Further time-of-flight secondary ion mass spectrometry (TOF-SIMS) analyses were conducted on both pristine and aged ZnO and AZO RS devices.

Overall, this study examines the shelf-aging characteristics of RS structures fabricated using electrodes and sol–gel-processed oxide materials commonly employed in QLEDs. Notably, we intentionally used an unstable sol–gel-processed ZnO and AZO, along with an asymmetric electrode structure of ITO/Al, which may underperform compared to a typical RS device fabricated by sputtering [17]. This choice emphasizes that the goal of this study is not to enhance RS device performance but rather to provide insights into the arbitrary aging phenomenon in QLEDs through combined research on QLEDs and RS devices.

## 2. Experimental Details

2-Methoxyethanol (99.8%), zinc acetate dihydrate (98%), aluminum nitrate nonahydrate (98%), and diethanolamine (99%) were purchased from Sigma-Aldrich. For the synthesis of the ZnO solution, 0.65 g of zinc acetate dihydrate was dissolved in 6.5 mL of 2-methoxyethanol with the dropwise addition of 0.179 mL of diethanolamine. The AZO solution was prepared following the same procedure as ZnO, with an additional 0.2219 g of aluminum nitrate nonahydrate added to the precursor, corresponding to a nominal Al/(Al+Zn) precursor metal-cation fraction of approximately 16.65%. All ZnO and AZO solutions were stirred at 1000 rpm overnight.

The RS devices had ITO/ZnO/Al and ITO/AZO/Al configurations (Figure 1b). Initially, an ITO-patterned glass substrate was sequentially sonicated in acetone and isopropanol for cleaning. It was then treated with ultraviolet ozone for over 10 min. Afterward, the ZnO or AZO sol–gel solutions were filtered through a 0.2 µm hydrophobic filter, deposited onto the substrate, and spin-coated at 2000 rpm for 40 s. Subsequently, the substrate underwent thermal annealing at 200 °C for 1 h in an ambient environment to induce hydrolysis reactions, forming the ZnO or AZO layer. Finally, an Al electrode with a thickness of over 100 nm was deposited by thermal evaporation at a pressure of 5 × 10^−6^ torr. The devices had a square active electrode-overlap region of 1.4 mm × 1.4 mm, corresponding to an area of 1.96 mm^2^. Cross-sectional TEM indicated an approximate ZnO thickness of 85 nm (Figure S1). AZO was prepared using the same spin-coating and thermal-treatment conditions and is expected to have a comparable thickness, although this expectation is based on the fabrication procedure rather than a direct AZO thickness measurement. XPS measurements were performed using an AXIS-HSi system (Kratos Analytical Ltd., Manchester, UK) with Al Kα radiation (1486.69 eV). Binding energies were referenced to the adventitious C 1s peak at 284.5 eV, and a Shirley background was used for peak fitting. The original pass-energy and detailed peak line-shape/constraint metadata could not be recovered from the archived analysis records. Optical constants were obtained by spectroscopic ellipsometry using an SE MG-1000 UV system (NANO-VIEW Co., Ltd., Ansan, Republic of Korea) at an incidence angle of 69.85°, with the spectra analyzed using a Tauc–Lorentz dispersion model.

Aging time zero was defined as the completion of device fabrication, and storage durations were counted from that point. Between electrical measurements, the unencapsulated devices were stored on a laboratory shelf in ambient air. The devices were exposed to the existing laboratory lighting without a prescribed illumination schedule and were not intentionally maintained in darkness. Storage temperature and relative humidity were neither independently controlled nor recorded, and oxygen partial pressure was not independently controlled. The aging series was obtained by repeatedly measuring the same devices at successive storage times; separate previously unmeasured devices were not used at each time point. Between electrical measurements, the devices were stored without an applied bias. Thus, the experimental history consisted of unbiased storage intervals interrupted by *I*–*V* characterization. No aged-but-unmeasured control group was included, and the measured evolution may therefore contain contributions from both storage and previous electrical characterization. Future studies using controlled-atmosphere storage and matched aged-but-unmeasured control devices will be required to separate storage-associated evolution from contributions of prior electrical characterization.

*I*–*V* measurements were conducted using a Keithley 2450 with a voltage increment of 0.02 V. No separate electroforming step was applied. A numerical operator-selected current-compliance setting is not recoverable from the archived measurement records. The dwell/integration time, sweep rate, and inter-sweep delay were likewise not retained and therefore cannot be reported reliably. Voltage was defined as the potential of Al relative to ITO. Positive and negative voltages therefore correspond to Al being positive and negative relative to ITO, respectively. For the representative *I*–*V* datasets used for the detailed curve and power-law analyses, ZnO was measured using 0 → +2 → −2 → 0 V, whereas AZO was measured using 0 → +3 → −3 → 0 V, in both cases with a 0.02 V voltage increment. The wider AZO voltage window was used to capture switching transitions extending above +2 V. Because the applied voltage excursion contributes to cumulative electrical stress and state preparation, differences in sweep window may also contribute to differences among the measured responses. SET and RESET were identified from clearly discernible abrupt increases and decreases in current magnitude associated with transitions to the lower- and higher-resistance branches, respectively. *V*_SET_ and *V*_RESET_ were assigned to the voltages at which these transitions were observed. TOF-SIMS measurements were performed using a TOF.SIMS 5 system (IONTOF GmbH, Münster, Germany) in positive-ion mode with charge compensation using a flood gun. A Bi1+ primary-ion beam (30 keV, 1 pA) rastered over 50 × 50 μm^2^ was used for analysis, while a Cs+ sputter beam (1 keV, 10 nA) rastered over 200 × 200 μm^2^ was used for depth profiling. One specimen was analyzed for each condition: pristine ZnO, shelf-aged ZnO, pristine AZO, and shelf-aged AZO. The specimens were dedicated to TOF-SIMS and were not electrically characterized before profiling. The exact elapsed storage durations of the aged TOF-SIMS specimens could not be reconstructed from the archived records. Secondary-ion signals are therefore presented as relative intensities and are not converted to absolute concentrations. Because the apparent sputtering time across corresponding layers differed among profiles, the time axes were relatively registered using the onset and decrease in the Zn-related signal, which defines the ZnO/AZO switching-layer region. This adjusted coordinate is referred to as compensated sputtering time. It is used only for qualitative profile alignment and does not represent calibrated physical depth.

## 3. Results and Discussion

The RS devices have energy levels as depicted in Figure 1c. This structure is also commonly used as an electron-only device (EOD) to evaluate electron injection and transport through ZnO [18]. ITO has a work function of approximately −4.7 eV, while Al has a work function of −4.2 eV. ZnO typically has an energy level of about −4.0 eV, and AZO is known to have a similar work function of −3.9 eV. The energy-level alignment provides qualitative context for carrier injection in the present structures; however, ideal ohmic injection at the ITO/oxide or oxide/Al contacts has not been independently established. Substitutional occupation of Zn sites is a commonly reported configuration for Al in ZnO; however, this specific configuration is not independently identified for all Al incorporated into the present AZO films. The configurations illustrated in Figure 1d,e therefore represent a possible structural model. Such substitutional incorporation, when it occurs, is facilitated by the smaller ionic radius of Al^3+^ (0.54 Å) compared with Zn^2+^ (0.74 Å) [19].

**Figure 2:**
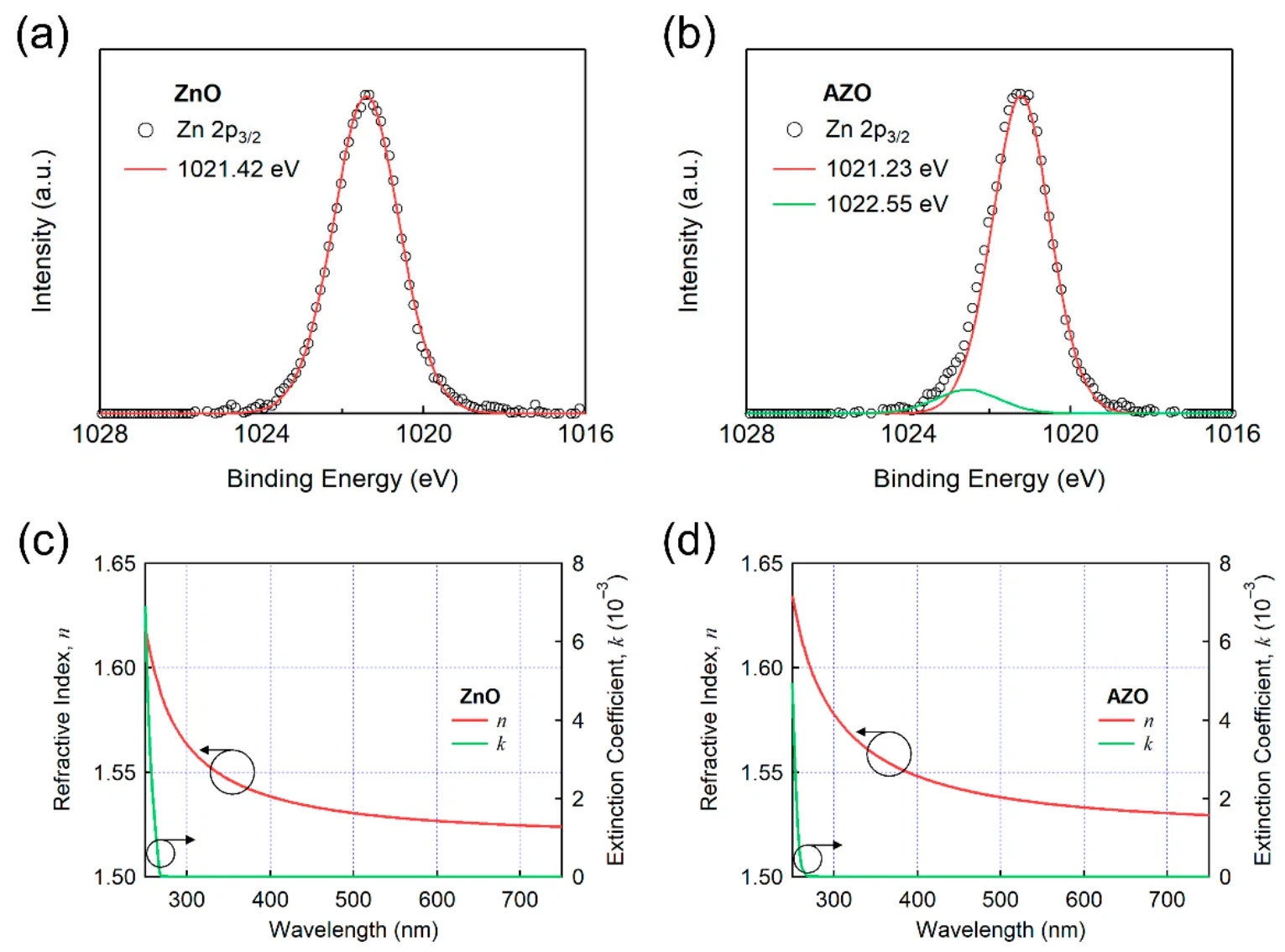
Deconvolution results of the XPS Zn peak for (**a**) ZnO and (**b**) AZO films. Refractive indices (***n***) and extinction coefficients (***k***) of (**c**) ZnO and (**d**) AZO films.

Particularly, the size difference causes lattice distortion and contraction, as the smaller Al^3+^ ions replace the larger Zn^2+^ ions [20]. In Figure 2a,b, the Zn 2p3/2 peak is located at 1021.42 eV. After Al addition, an additional feature with a separation of approximately 1.32 eV is observed. Similar changes in the Zn 2p3/2 spectrum have been reported for Al-doped ZnO and are associated with Al incorporation into ZnO [21]. Therefore, the present spectral change is considered consistent with Al incorporation, although it does not by itself uniquely confirm substitutional occupation of Al at Zn sites. Al incorporation can modify the electronic and optical properties of ZnO. Figure 2c,d shows differences in the optical constants of the ZnO and AZO films. However, despite the increase in *n* with Al doping, sol–gel-processed ZnO and AZO films exhibit lower *n* than those produced by methods such as physical or chemical vapor deposition [22,23]. This difference arises from microstructural variations, primarily due to the lower density of films produced via the sol–gel method [24]. The sol–gel process tends to produce films with a more porous structure and higher levels of structural imperfections compared to other deposition techniques.

**Figure 3:**
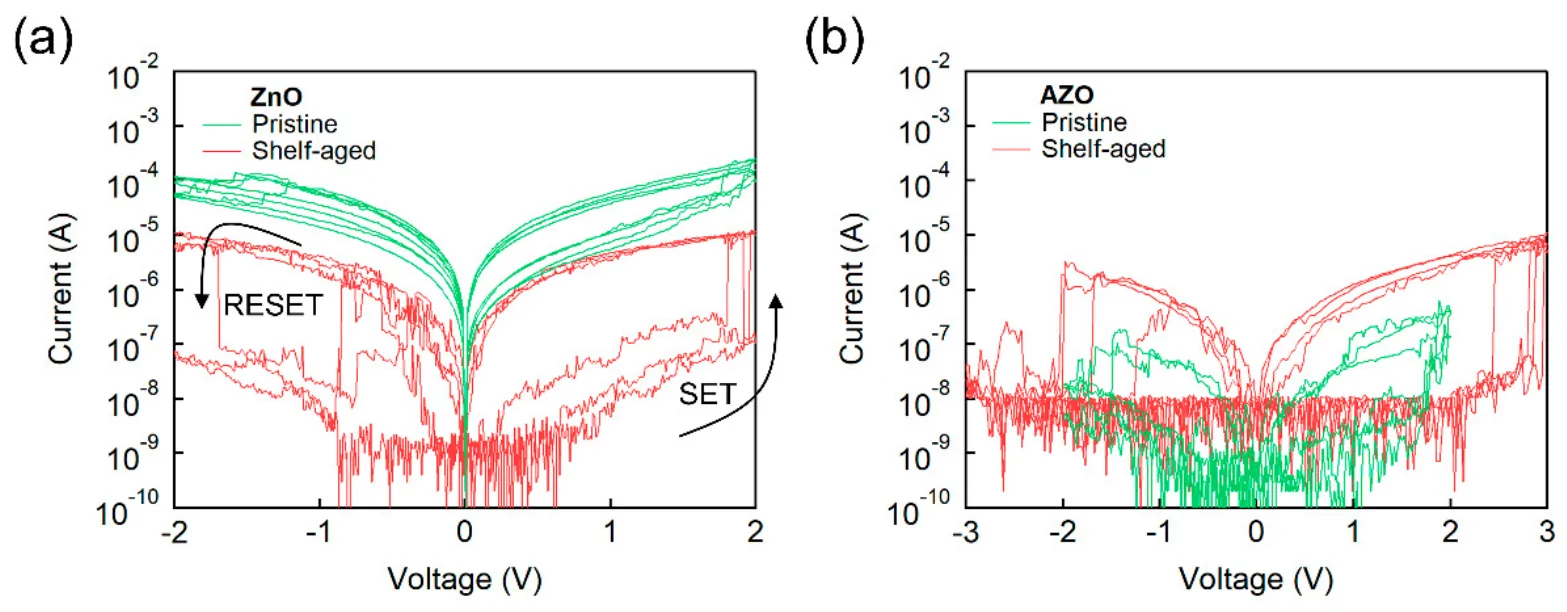
Representative *I*–*V* characteristics of pristine and shelf-aged RS devices with (**a**) ZnO and (**b**) AZO dielectric layers.

Figure 3 displays the RS characteristics of devices based on ZnO and AZO, highlighting several notable observations. For Figure 3, the pristine ZnO dataset contains one sweep from each of four different cells on the same substrate, whereas the shelf-aged ZnO dataset contains three repeated sweeps from one cell. The pristine AZO dataset contains three repeated sweeps from one cell, and the shelf-aged AZO dataset contains four repeated sweeps from one cell. The pristine ZnO and AZO measurements shown in Figure 3 were performed approximately 1 day after fabrication, whereas the shelf-aged ZnO and AZO measurements were obtained approximately 22 and 8 days after fabrication, respectively. Initially, the pristine ZnO device exhibits a *V*_SET_ of 1.8–1.9 V. At a read voltage (*V*_READ_) of 1 V, the current in LRS is denoted as *I*_on_, while the current in HRS is denoted as *I*_off_. In the pristine ZnO device, *I*_off_ ranges between 5.43 × 10^−6^ and 1.20 × 10^−5^, while *I*_on_ ranges between 3.78 × 10^−5^ and 5.99 × 10^−5^, resulting in an *I*_on_/*I*_off_ ratio of 4.13–8.20. In contrast, the shelf-aged ZnO device shown in Figure 3a exhibits similar *V*_SET_ voltages, but with a significantly increased *I*_on_/*I*_off_ ratio. *I*_off_ ranges between 4.50 × 10^−9^ and 4.43 × 10^−8^, while *I*_on_ ranges between 3.57 × 10^−6^ and 4.71 × 10^−6^, yielding an *I*_on_/*I*_off_ ratio of 8.07 × 10^1^ to 1.05 × 10^3^. This change highlights several key points: First, there is a dramatic reduction in current levels. *I*_off_ has decreased by over three orders of magnitude, from 10^−6^ to 10^−5^ A to 10^−9^ to 10^−8^ A, while *I*_on_ has decreased by one order, from 10^−5^ to 10^−6^ A. Several factors may contribute to this behavior in ZnO devices. One possible contribution is Al redistribution from the Al electrode into ZnO, which has been reported in Al/ZnO/Al RRAM structures [25]. Additionally, Al diffusion and interfacial AlO*_x_* formation have also been reported in Al/ZnO/Pt RRAM devices [26]. A detailed analysis of these effects will be presented in a later section.

Next, examining the characteristics of the AZO device before and after shelf-aging, the pristine device exhibits a *V*_SET_ comparable to that of the ZnO device, ranging between 1.7–1.8 V. For the representative pristine AZO measurements shown in Figure 3b, *I*_off_ ranges from 1.30 × 10^−9^ to 4.80 × 10^−9^ A and Ion ranges from 4.69 × 10^−8^ to 1.46 × 10^−7^ A at *V*_READ_ = 1 V, corresponding to an *I*_on_/*I*_off_ range of approximately 10.8–112. In addition, when comparing the characteristics of pristine ZnO and AZO devices, AZO exhibits a significantly lower current order than ZnO, primarily due to the impact of Al doping. Typically, doping ZnO with Al results in substitutional doping where Al^3+^ replaces Zn^2+^, leading to an increase in the concentration of additional free electrons, enhancing mobility and conductivity [27]. However, it can also potentially decrease mobility or conductivity due to several factors. First, overdoping may lead to defect formation and impurity states that act as scattering centers or trap charge carriers, reducing their availability for conduction [28]. Additionally, lattice strain from size mismatch between Al and Zn ions, inhomogeneous dopant distribution, and altered grain boundary properties can further impair carrier mobility and electrical performance [29]. Indeed, various studies on optoelectronic applications have reported reduced conductivity and electron transport capabilities in AZO compared to pristine ZnO [30]. The shelf-aged AZO device shows a higher *V*_SET_ of 2.4–2.9 V, with *I*_off_ between 7.50 × 10^−9^ and 1.04 × 10^−8^ and *I*_on_ between 4.50 × 10^−7^ and 1.26 × 10^−6^, resulting in an *I*_on_/*I*_off_ ratio from 6.00 × 10^1^ to 1.23 × 10^2^. While pristine ZnO and AZO devices initially exhibited distinct differences in current orders depending on doping, they gradually converged to a similar current level with shelf-aging. Overall, the increase in current level and *I*_on_/*I*_off_ ratio of the AZO device upon shelf-aging may reflect changes in oxygen-related defect states and interfacial charge injections, as discussed below.

**Figure 4:**
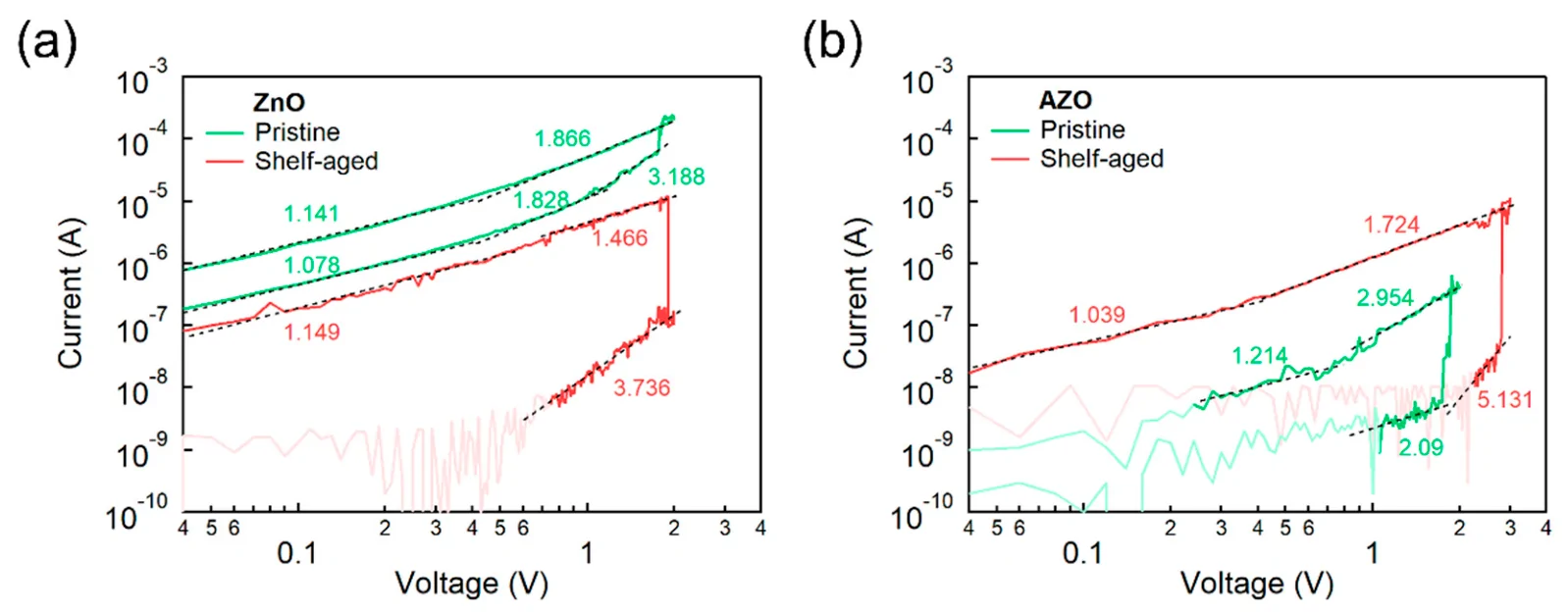
Log–log scale *I*–*V* characteristics of pristine and shelf-aged RS devices with (**a**) ZnO and (**b**) AZO dielectric layers. The fitting intervals, sweep directions, resistance states, slopes, slope standard errors, and R^2^ values are summarized in Table S1.

The *I*–*V* characteristics are examined on a log–log scale to identify voltage-dependent power-law behavior and assess its compatibility with possible conduction mechanisms (Figure 4). The individual log(|*I*|)–log(|*V*|) segments were fitted using log(|*I*|) = *m* log(|*V*|) + *b*, where *m* is the fitted power-law exponent. The fitting ranges, numbers of data points, fitted slopes, slope standard errors, and R^2^ values are provided in Table S1. These statistics characterize the quality of the power-law fits within the selected intervals and do not independently verify a unique microscopic conduction mechanism. In brief, the equation $J=\mathrm{qn\mu}\frac{V}{d}$ describes ohmic conduction, where *J* represents the current density, *q* is the elementary charge of an electron, *n* is the number of charge carriers per unit volume, *μ* is the mobility of the charge carriers, *V* is the applied voltage, and *d* is the distance between the electrodes. This relationship reflects the behavior of many conductors under low $\vec{E}$, where the current is a linear response to the voltage. However, as the applied voltage increases, the conduction mechanism begins to be governed by an alternative process. The Mott–Gurney relation, $J=\frac{9}{8}\varepsilon_{0}\varepsilon_{r}\mu \frac{V^{2}}{d^{3}}$, describes ideal SCLC under specific assumptions, including approximately ohmic injection and uniform bulk transport [31]. Figure 4 presents measured current rather than current density; however, normalization by the constant device area changes only the intercept of the log–log plot and not the fitted slope. Because the assumptions of the ideal model have not been independently established here, the fitted exponents are used primarily to characterize the observed voltage dependence rather than to uniquely identify a conduction mechanism. This form of current is quadratic with respect to the applied voltage and is typical in materials where the injection of charge carriers at the electrodes leads to the formation of a significant space-charge region, influencing $\vec{E}$ distribution and, consequently, the current through the material.

Following the understanding of SCLC, another critical dynamic in charge conduction is the trap-controlled space-charge-limited current (TC-SCLC) [32]. In TC-SCLC, the current is not solely dictated by the bulk properties of the intrinsic material, but also by the presence, density, and energy states of traps. These traps can capture charge carriers, leading to a modification of the internal electric field ($\vec{E}$) and a reduction in the overall mobility of carriers. As a result, the *I*–*V* relationship deviates from the simple square law observed in pure SCLC scenarios, often showing a more complex, non-linear behavior that depends on the trap dynamics, such as the rates of trapping and de-trapping. This model is crucial for designing and understanding devices based on materials with significant levels of impurities or defects such as oxygen vacancies, as it allows for a more accurate prediction of their electrical performance under various operating conditions [32]. At *V*_TC_, defined here as the voltage at which the current begins to increase with a much steeper slope, the transport may enter a regime in which trap-related processes become increasingly important. TC-SCLC provides one possible framework for interpreting superlinear *I*–*V* behavior in defect-rich materials [32]; however, the present data do not uniquely establish this regime or link it directly to conductive-filament dynamics.

For pristine ZnO (Figure 4a), the forward-sweep HRS regions of 0.04–0.26 V, 0.60–0.92 V, and 1.18–1.68 V yield slopes of 1.078 ± 0.013, 1.828 ± 0.037, and 3.188 ± 0.117, respectively, with corresponding R^2^ values of 0.9986, 0.9940, and 0.9689. During the reverse sweep, the LRS regions of 0.72–1.78 V and 0.04–0.26 V yield slopes of 1.866 ± 0.013 and 1.141 ± 0.020, with R^2^ values of 0.9975 and 0.9968, respectively. For shelf-aged ZnO, the forward-sweep HRS region of 0.76–1.88 V yields a slope of 3.736 ± 0.074 (R^2^ = 0.9587), whereas the reverse-sweep LRS regions of 0.78–1.82 V and 0.04–0.49 V yield slopes of 1.466 ± 0.027 (R^2^ = 0.9667) and 1.149 ± 0.029 (R^2^ = 0.9736), respectively. For pristine AZO (Figure 4b), the retained forward-sweep HRS region of 1.06–1.64 V yields a slope of 2.090 ± 0.377, although the corresponding R^2^ of 0.5227 indicates that this interval is only weakly described by a single power-law relationship. During the reverse sweep, the 0.94–1.82 V and 0.26–0.72 V LRS regions yield slopes of 2.954 ± 0.076 and 1.214 ± 0.140, with R^2^ values of 0.9722 and 0.7737, respectively. For shelf-aged AZO, the forward-sweep HRS region of 2.34–2.76 V yields a slope of 5.131 ± 1.272 with R^2^ = 0.5557, indicating considerable deviation from a single linear dependence in log–log coordinates. During the reverse sweep, the 0.51–2.01 V and 0.06–0.33 V LRS regions yield slopes of 1.724 ± 0.006 and 1.039 ± 0.048, with R^2^ values of 0.9995 and 0.9833, respectively. The fitted exponents quantitatively describe the voltage dependence within the selected intervals. Slopes close to unity are consistent with approximately ohmic-like behavior, whereas larger exponents indicate increasingly superlinear transport. However, a particular exponent does not by itself uniquely identify SCLC or trap-controlled SCLC, particularly for intervals with relatively low R^2^ values.

Several trends are evident in the pristine and shelf-aged *I*–*V* curves of the ZnO and AZO devices. The nonlinear *I*–*V* characteristics suggest a possible contribution from defect-assisted transport. The observed hysteresis and nonlinear *I*–*V* characteristics support resistive-switching behavior but do not by themselves establish oxygen-vacancy filament formation. Oxygen-related defect states may contribute to this behavior; however, the present measurements do not directly determine the oxygen-vacancy concentration or uniquely identify the microscopic origin of the traps. Within a proposed oxygen-defect-mediated switching model, electrical bias may promote redistribution of oxygen-related species and modify conductive pathways. The fitted exponents and voltage dependence change after shelf-aging. These changes may reflect altered trapping or injection conditions. Alternative transport mechanisms, including interface-limited transport, Poole–Frenkel emission, hopping, and tunneling, cannot be excluded based on the present measurements. Such changes in voltage dependence may reflect altered trapping or injection conditions and may influence the stability of the resistive states.

**Figure 5:**
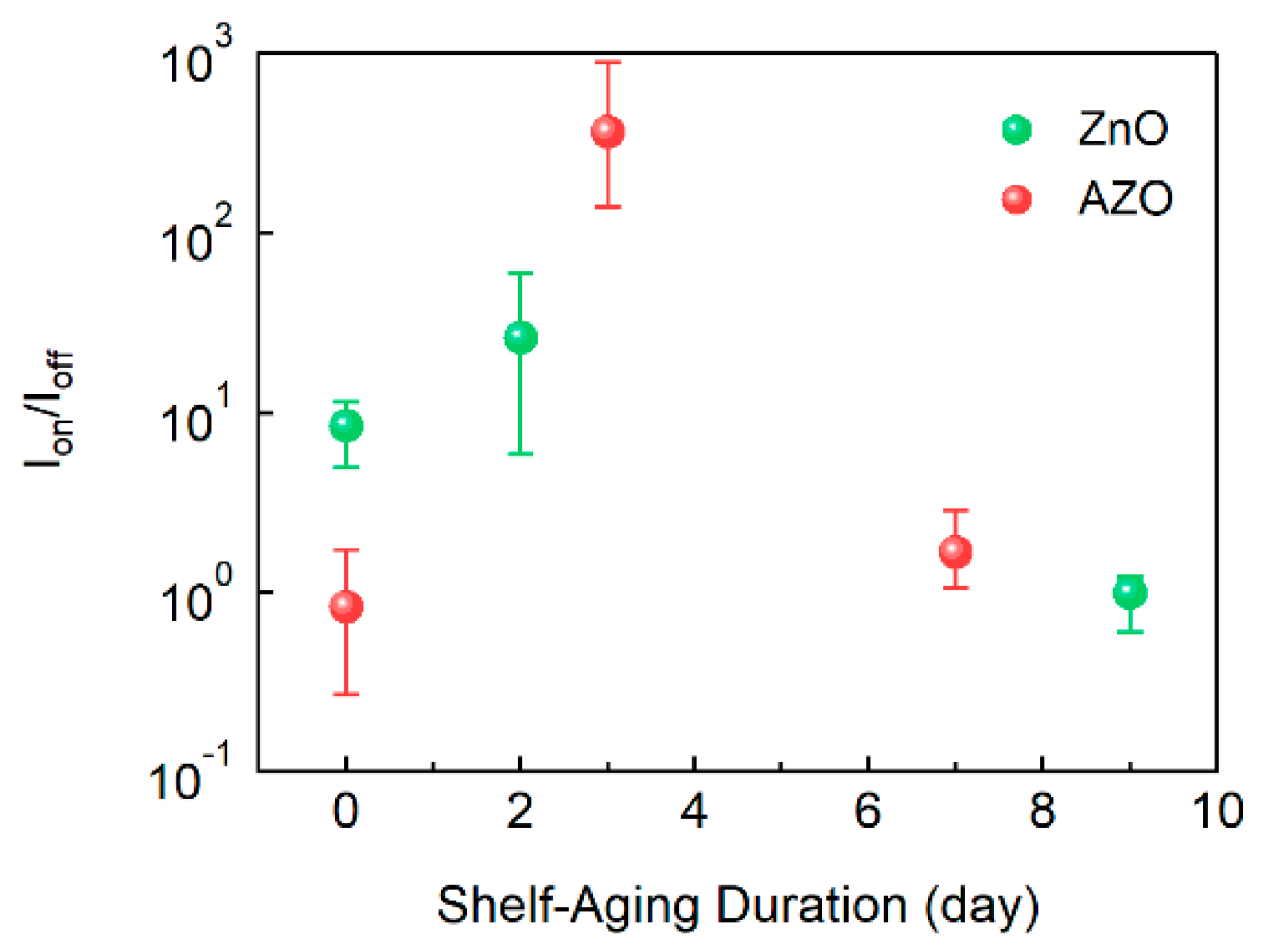
Evolution of the *I*_on_/*I*_off_ ratio in ZnO and AZO RS devices as a function of shelf-aging time after fabrication. The data were obtained from a longitudinal aging series measured at successive storage times. The number of retained ratio measurements at each time point is provided in Table S2; complete cell-level identifiers for the individual measurements could not be reconstructed from the archived summary. *I*_on_/*I*_off_ was evaluated at *V*_READ_ = 1 V. Here, *I*_on_ and *I*_off_ denote fixed sweep-branch labels retained from the original analysis rather than currents reassigned according to the instantaneous LRS/HRS ordering. Markers represent arithmetic means of the recorded values, and error bars indicate the observed minimum-to-maximum range.

Additionally, Figure 5 illustrates changes in the *I*_on_/*I*_off_ ratio over the duration of shelf-aging. Notably, there is a significant variation in the *I*_on_/*I*_off_ ratio with aging, which is a key characteristic of RS devices. In brief, the *I*_on_/*I*_off_ ratio in RS devices is crucial as it determines the device’s ability to clearly distinguish between different resistance states, typically representing ‘on’ (LRS) and ‘off’ (HRS) states. This ratio is a key parameter influencing device performance in terms of data storage capacity, signal-to-noise ratio, and overall reliability [33,34]. A higher *I*_on_/*I*_off_ ratio provides greater current contrast between HRS and LRS. In the present study, however, the observed changes in *I*_on_/*I*_off_ are not interpreted as direct evidence of improved endurance or operational lifetime. We see that initially, in their pristine states, both ZnO and AZO devices have *I*_on_/*I*_off_ ratios of less than 10. To clarify, all measurements in Figure 5 are based on a *V*_READ_ of 1 V. Figure 5 was obtained from a fabrication run separate from that used for Figure 3 and represents a longitudinal aging series. The retained Figure 5 dataset contains 5, 3, and 4 recorded ratios for ZnO at 0, 2, and 9 days, respectively, and 3, 7, and 4 recorded ratios for AZO at 0, 3, and 7 days, respectively. Complete cell identifiers for the Figure 5 measurements are not preserved in the archived summary; therefore, these measurement counts are not interpreted as independent-device counts or population-level device statistics. The archived Figure 5 summary does not retain the sweep-window metadata for every individual contributing measurement; therefore, storage-associated evolution cannot be quantitatively separated from possible differences in prior electrical stress. Each data point in Figure 5 represents the arithmetic mean of the recorded *I*_on_/*I*_off_ values at the corresponding storage time, and the error bars indicate the observed minimum-to-maximum range. For the Figure 5 analysis, *I*_on_ and *I*_off_ are retained as operational labels for the two fixed sweep branches used in the original analysis rather than being reassigned according to their instantaneous current magnitudes. Accordingly, *I*_on_/*I*_off_ in Figure 5 represents a fixed branch-current ratio, and values below unity can occur when the two branches overlap or cross as the switching contrast becomes weak. The ZnO device showed an increase in the mean recorded current ratio from 8.52 at day 0 to 26.3 after 2 days of shelf-aging. Similarly, the AZO device exhibited an increase from approximately 1 in the pristine state to 368 after 3 days of shelf-aging. However, at the later measured storage times—9 days for ZnO and 7 days for AZO—both devices exhibited a substantial decrease in *I*_on_/*I*_off_. After 9 days of shelf-aging, the ZnO device transitioned to a state with almost no hysteresis, with the ratio approaching 1. Similarly, the AZO device experienced a dramatic decline in *I*_on_/*I*_off_, dropping to 1.67 after 7 days compared to 368 after 3 days. A nonmonotonic evolution of switching contrast was observed within the investigated storage periods of up to nine days for ZnO and seven days for AZO. The present results do not determine whether the switching window continues to decrease, stabilizes, or partially recovers during longer storage. Furthermore, an *I*_on_/*I*_off_ ratio approaching unity does not by itself determine irreversible electrical failure. Longer storage and recovery measurements would be required to determine the persistence and reversibility of the degraded response.

Nonetheless, the reasons behind the increase and subsequent decrease in the *I*_on_/*I*_off_ ratio due to shelf-aging can be understood through insights from QLED research. The proposed shelf-aging mechanism in QLEDs involves both positive aging, which enhances device characteristics, and negative aging, which degrades them, as shown in Figure 1a. For instance, a previous report observed that in a QLED with an ITO/PEDOT:PSS/TFB/CdSe/ZnMgO/Al structure, the external quantum efficiency of a blue QLED increased from 4.93% to 12.97%, representing a 2.63-fold increase after approximately 5–8 days of shelf-aging [14]. The improvement was attributed to two main factors. First, the formation of an AlO*_x_* layer at the ZnMgO/Al interface passivated defect states on the ZnMgO surface, reducing trap-assisted nonradiative recombination. Second, the escape of oxygen and the resulting increase in oxygen vacancies during AlO*_x_* formation enhanced electron conductivity and optimized the electron–hole injection balance, leading to improved device efficiency. Similarly, the formation of an AlO*_x_* layer and variations in oxygen vacancies in the ZnO/Al structure due to shelf-aging have been proposed in a few QLED-related studies [35]. More broadly, interfacial engineering in oxide-based hybrid materials has also been shown to modify charge-transport behavior, as exemplified by MnCo_2_O_4_/PANI nanohybrids [36]. Reported QLED aging studies show electrical or efficiency changes over study-dependent day-scale storage periods (Table 1) [11,13,14,15,37]. In the present sampled aging series, the highest measured *I*_on_/*I*_off_ values occurred after 2 days for ZnO and 3 days for AZO, followed by substantially lower values at the later measured storage times. Importantly, we observed that comparable day-scale shelf-aging effects occur in RS devices constructed solely from components commonly used in QLEDs, such as the ZnO dielectric layer and Al top electrode, without being fully fabricated QLED devices. The occurrence of electrical changes over a comparable day-scale motivates comparison with reported QLED aging studies but does not provide direct evidence for a common microscopic mechanism. A possible relationship between evolving conductive pathways in oxide-based structures and QLED aging therefore remains a hypothesis for future testing.

**Figure 6:**
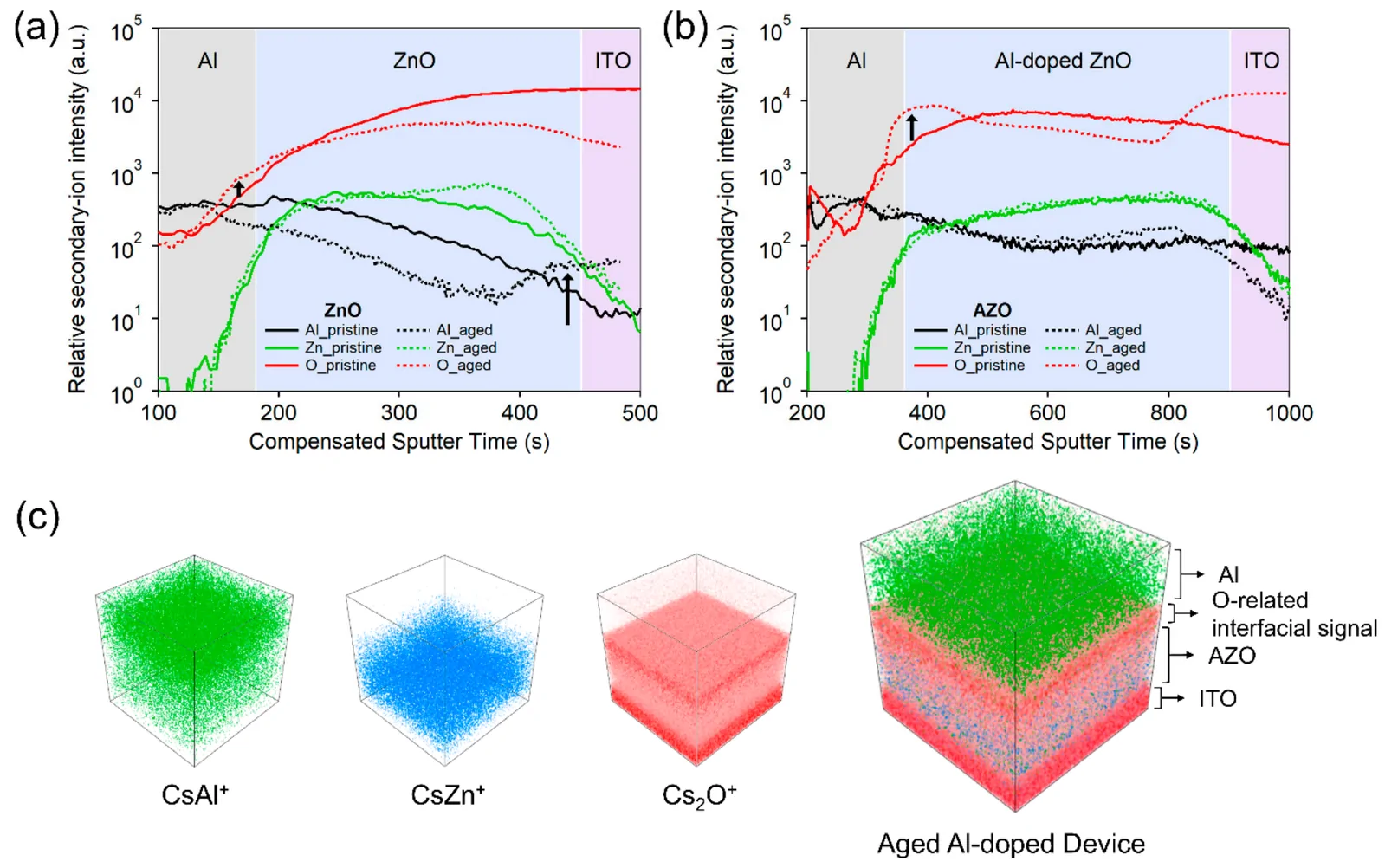
Qualitative TOF-SIMS secondary-ion profiles of pristine and shelf-aged devices containing (**a**) ZnO and (**b**) AZO, plotted as relative secondary-ion intensity versus compensated sputtering time. The profiles do not represent calibrated elemental concentrations or physical-depth distributions. (**c**) Three-dimensional visualization of the detected secondary-ion distributions in the shelf-aged AZO device, showing an O-related signal near the Al/AZO transition. The arrows indicate the positions along the sputtering-time axis and the relative magnitude of the increased oxygen-related signal.

Figure 6 compares the TOF-SIMS profiles of pristine and archived aged ZnO and AZO specimens to examine qualitative differences in the secondary-ion distributions. Because the exact storage durations of the aged TOF-SIMS specimens were not preserved, these qualitative profiles cannot be quantitatively assigned to the 2-, 3-, 7-, or 9-day electrical-aging points. An enhanced oxygen-related signal is observed near the dielectric/Al interface after shelf-aging. This observation is consistent with a change in interfacial oxygen-containing species, potentially including AlO*_x_*. However, the present TOF-SIMS signal does not directly identify the chemical bonding environment of oxygen and therefore does not uniquely prove AlO*_x_* formation. Interfacial Al oxidation has been reported for a related ZnMgO/Al interface using chemical-state-sensitive XPS measurements [14]. This literature result supports considering Al oxidation as a possible interfacial process in the present structures. Such interfacial oxidation, when present, may affect charge transport in transition-metal-oxide thin films by modifying surface or interfacial defect states and carrier injection [14]. In the ZnO device, the reduced current level after shelf-aging may therefore reflect changes in interfacial passivation, charge injection, or oxygen-related defect states. The relative oxygen-vacancy concentrations of the present ZnO and AZO films were not directly measured. For the AZO device, the increased LRS current after shelf-aging may reflect changes in oxygen-related defect states, interfacial charge injection, or both. An aging-induced increase in oxygen-vacancy concentration remains a possible interpretation rather than an experimentally established result.

Figure 6a shows an aging-associated increase in the Al-related secondary-ion signal in the region assigned to the Al electrode and a decrease near the region assigned to the ITO/ZnO interface, whereas the AZO profiles exhibit a more pronounced change (Figure 6b). In brief, the relative secondary-ion profiles do not solely identify saturation of substitutional sites or a corresponding reduction in Al diffusivity. Furthermore, the Al-related signal in AZO does not distinguish Al originating from the top electrode from Al initially incorporated into the dielectric. The profiles are interpreted qualitatively rather than as calibrated concentration–depth distributions. Sputter-induced mixing, interface roughness, and matrix-dependent secondary-ion yields may influence the apparent distributions [38]. Accordingly, no Al diffusion coefficient, diffusion length, or absolute concentration is extracted from these measurements. Nonetheless, the AZO results are consistent with aging-associated interfacial evolution, potentially involving changes in oxygen-containing species near the AZO/Al interface (Figure 6c). As discussed previously, *V*_SET_ increased from 1.7–1.8 V in the pristine state to 2.4–2.9 V after shelf-aging (Figure 3b), which might reflect changes in the interfacial barrier or switching pathway. At the later measured storage times, the reduced switching window may reflect changes in oxygen-related defect states, interfacial barriers, or the reversibility of conductive pathways. Within a proposed filamentary interpretation, an imbalance between conductive-path formation and disruption could impair RESET behavior.

**Figure 7:**
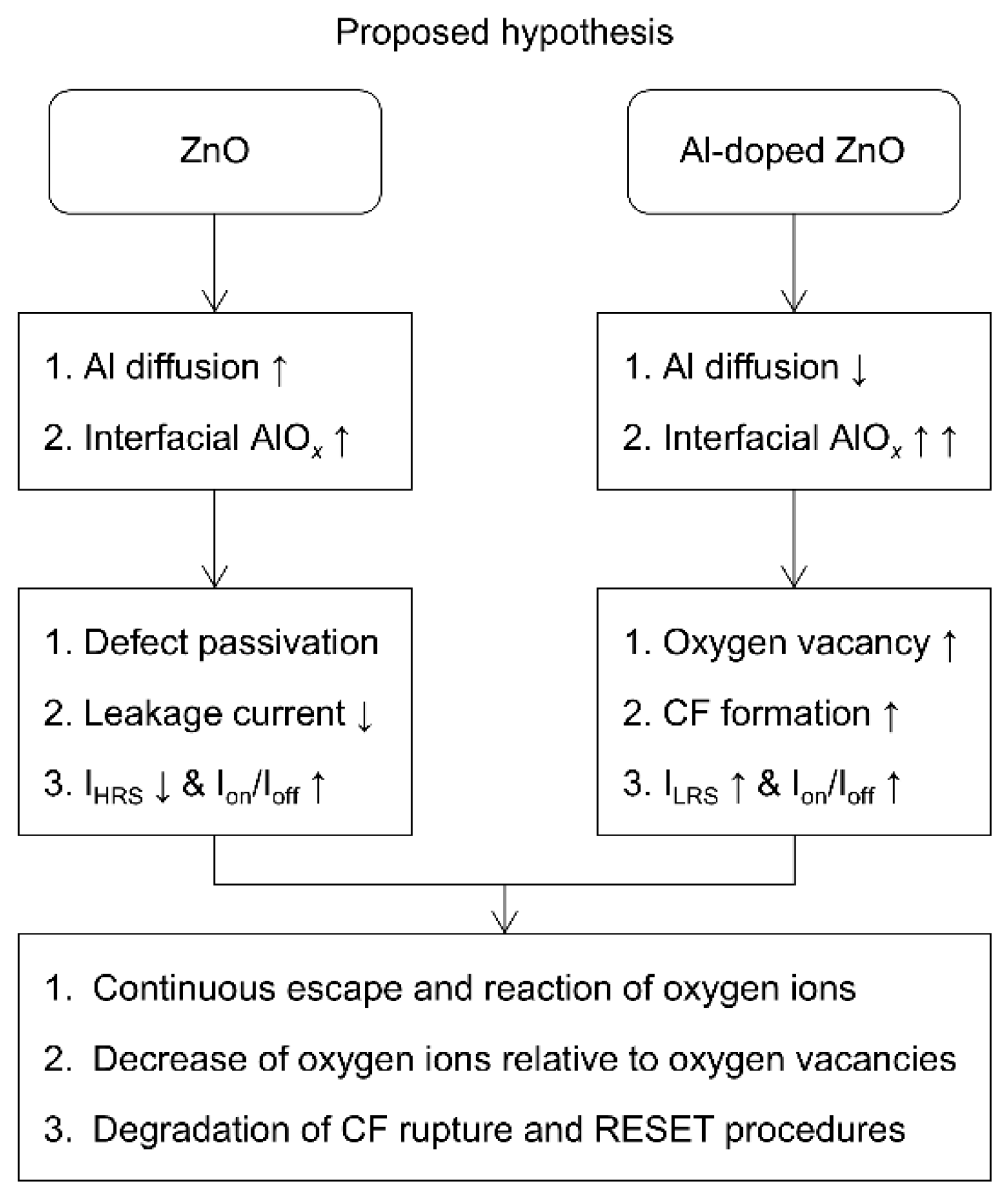
Proposed mechanistic hypothesis for the shelf-aging behavior of ZnO and AZO RS devices. The illustrated interfacial reactions, oxygen-related defect processes, and conductive-pathway evolution represent possible interpretations of the experimental observations rather than directly measured microscopic processes.

Finally, Figure 7 presents a proposed mechanistic interpretation of the observed shelf-aging behavior. Interfacial oxidation, changes in oxygen-related defect states, surface or interfacial passivation, and evolution of conductive pathways may contribute to the different electrical responses of ZnO and AZO devices. However, the present measurements do not directly establish oxygen-vacancy evolution, defect passivation, oxygen-ion depletion, or a unique sequence of filament formation and rupture. The increases and decreases in Al diffusion indicated in Figure 7 are also unverified assumptions within the proposed mechanism, as the present measurements do not determine the direction or extent of Al redistribution. The illustrated processes should therefore be regarded as possible microscopic mechanisms rather than experimentally confirmed reactions.

## 4. Conclusions

We investigated the electrical evolution of ITO/ZnO/Al and ITO/AZO/Al RS structures during ambient storage with repeated electrical measurements. The results characterize changes in switching contrast and nonlinear conduction in these simplified structures and provide a basis for considering possible interfacial and defect-related processes. In the present measurements, the highest *I*_on_/*I*_off_ values were observed after two days of storage for ZnO and three days for AZO, followed by a decrease at later storage times. The *I*–*V* analysis revealed changes in nonlinear conduction and RS behavior following shelf-aging. Fitted power-law exponents characterize the voltage dependence within the analyzed intervals. TOF-SIMS analyses revealed qualitative differences in the secondary-ion profiles of pristine and archived aged ZnO and AZO specimens. Because the exact storage durations of the aged specimens were not preserved, these profiles cannot be quantitatively associated with specific electrical-aging time points. Interfacial oxidation, oxygen-related defect changes, and altered charge injection remain possible contributors to the observed electrical evolution. Following the initial improvement, the devices examined in the aging series exhibited a substantially reduced switching window at the later measured storage times. Changes in oxygen-related defects, interfacial transport, or conductive-pathway reversibility may contribute to this deterioration, but their specific roles remain to be further studied.

To conclude, this study examined shelf-aging in sol–gel-processed ZnO- and AZO-based RS structures with Al electrodes from an optoelectronic perspective. The present results document electrical changes with elapsed storage time together with qualitative secondary-ion profile differences between pristine and archived aged ZnO and AZO specimens. Because the present structures do not reproduce the multilayer architecture, encapsulation, carrier balance, or electroluminescent response of complete QLEDs, validation of any connection to QLED-positive aging will require further experiments on complete QLEDs under defined storage and operating conditions.

## Figures and Tables

**Table 1:** Contextual comparison of device architecture, storage conditions, encapsulation, electrical history, aging time scale, experimentally observed responses, and proposed mechanistic interpretations in QLED-related and RS studies.

Device Type	Ref.	Device Structure	Atmosphere	Encapsulation	Electrical Stress/History	Aging Time Scale	Measured Response	Proposed Mechanism/Interpretation
QLED	[11]	ITO/PEDOT:PSS/TFB/CdSe/ZnO/Al	Glovebox storage, measurements in ambient air	UV-curable resin	Unreported	1, 3, 7 days	Positive aging during shelf storage	Interaction of resin components with ZnO; surface passivation/defect reduction
	[37]	ITO/PEDOT:PSS/TFB/InP/ZnMgO/Al	N_2_-filled glovebox	Epoxy	Unreported; post-annealing investigated	4 days	Positive aging in epoxy-encapsulated InP QD-LEDs	Thiol-related interaction/diffusion from epoxy and post-annealing effects
	[14]	ITO/PEDOT:PSS/TFB/CdSe/ZnMgO/Al	N_2_-filled glovebox	UV-curable resin	A new previously untested device was measured at each storage time	0, 3, 5, 8 days	EQE increase from 4.93% to 12.97% after shelf aging	Interfacial AlO*_x_* formation; surface-defect passivation; proposed increase in oxygen vacancies; improved electron–hole balance
	[13]	ITO/PEDOT:PSS/CdSe/ZnO/Al	N_2_ glovebox, wet/inert ambient experiments	UV-curable resin	Unreported	4, 6, 8, 27 days	Positive-aging response associated with ZnO surface evolution	–OH-related surface stabilization and reduced oxygen-species adsorption on ZnO
QLED + RS test structure	[15]	ITO/PEDOT:PSS/TFB/CdSe QDs/ZnMgO/(PVP)/Al; separate ITO/ZnMgO/Al RS device	Unreported	Unreported	Unreported	10 days	Aging behavior associated with RS characteristics in ZnMgO	O^2−^ redistribution/stacking; QD oxidation; proposed CF formation and rupture affecting device performance
Dedicated RS structure	This work	ITO/ZnO/Al and ITO/AZO/Al	Ambient laboratory air; T/RH/O_2_ partial pressure uncontrolled	Unencapsulated	Intermittent bipolar I–V characterization; unbiased storage between measurements	ZnO: 0, 2, 9 days; AZO: 0, 3, 7 days	Electrical switching evolution, fixed branch-current ratio, switching parameters; qualitative TOF-SIMS comparison	Interfacial oxidation, oxygen-related defect evolution, and conductive-pathway changes proposed as hypotheses only

## Data Availability

The original contributions presented in this study are included in the article and Supplementary Material. Further inquiries can be directed to the corresponding author.

## Supplementary Materials

The following supporting information can be downloaded at https://www.mdpi.com/article/10.3390/nano16191263/s1: Figure S1: Cross-sectional TEM image of the ZnO-based device; Table S1: Linear regression results for the log(I)–log(V) segments presented in Figure 4; Table S2: Individual fixed branch-current ratios used to construct Figure 5.
