# Supplementary material for: Influence of Shelf-Aging on Resistive Switching Characteristics of Sol–Gel-Processed ZnO and Al-Doped ZnO Structures
Source: Nanomaterials (Basel). 2026 Oct 7;16(19):1263. doi: 10.3390/nano16191263 (PMC13666779; doi:10.3390/nano16191263)
Supplement: Supplementary file 1 [file nanomaterials-16-01263-s001.zip › nanomaterials-4566409-supplementary.pdf]

Supporting Information

Influence of Shelf-Aging on Resistive Switching Characteristics of Sol-Gel-Processed ZnO and Al-Doped ZnO Structures

Dongjin Moon and Jaehoon Kim*

Department of Semiconductor Engineering, Gachon University, Seongnam-si, Gyeonggi-do, 13120, Republic of Korea

E-mail address: jaehoonkim@gachon.ac.kr

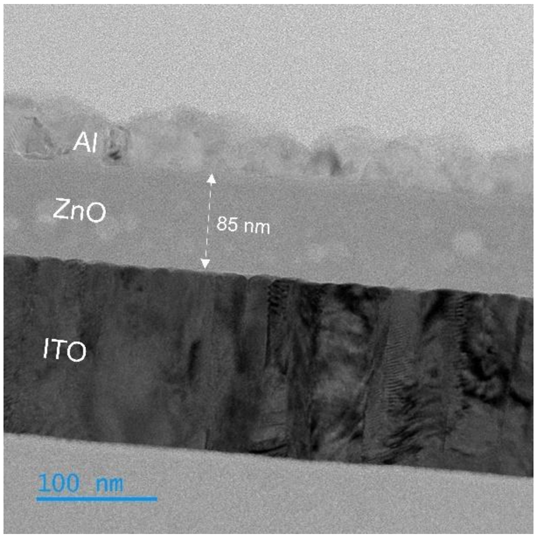

Figure S1. Cross-sectional TEM image of the ZnO-based device, showing an approximate ZnO layer thickness of 85 nm.

Table S1. Linear regression results for the log(*I*)–log(*V*) segments presented in **Figure 4**. Slope uncertainties are reported as standard errors. The noisy low-current region below 10^{-9} A was excluded from quantitative fitting and conduction-mechanism assignment.

Device condition	Sweep	State	Fitting range (V)	N	Slope	SE of slope	R^2
Pristine ZnO	Forward	HRS	0.04–0.26	12	1.078	0.013	0.9986
Pristine ZnO	Forward	HRS	0.60–0.92	17	1.828	0.037	0.994
Pristine ZnO	Forward	HRS	1.18–1.68	26	3.188	0.117	0.9689
Pristine ZnO	Reverse	LRS	0.72–1.78	54	1.866	0.013	0.9975
Pristine ZnO	Reverse	LRS	0.04–0.26	12	1.141	0.02	0.9968
Shelf-aged ZnO	Forward	HRS	0.76–1.88	113	3.736	0.074	0.9587
Shelf-aged ZnO	Reverse	LRS	0.78–1.82	105	1.466	0.027	0.9667
Shelf-aged ZnO	Reverse	LRS	0.04–0.49	46	1.149	0.029	0.9736
Pristine AZO	Forward	HRS	1.06–1.64	30	2.09	0.377	0.5227
Pristine AZO	Reverse	LRS	0.94–1.82	45	2.954	0.076	0.9722
Pristine AZO	Reverse	LRS	0.26–0.72	24	1.214	0.14	0.7737
Shelf-aged AZO	Forward	HRS	2.34–2.76	15	5.131	1.272	0.5557
Shelf-aged AZO	Reverse	LRS	0.51–2.01	51	1.724	0.006	0.9995
Shelf-aged AZO	Reverse	LRS	0.06–0.33	10	1.039	0.048	0.9833

Note: Linear regression was performed in $\log_{10}(|I|)$–$\log_{10}(|V|)$ coordinates. The reported uncertainty represents the standard error (SE) of the fitted slope. R^2 was calculated in logarithmic coordinates and describes the goodness of the linear fit within each selected voltage interval. The fitting intervals were selected as descriptive segments of the measured I–V curves rather than as independently established transport regimes. The fitted exponents are used to characterize the voltage dependence and do not, by themselves, uniquely establish a specific microscopic conduction mechanism.

Table S2. Individual fixed branch-current ratios (I_{on}/I_{off}, using the branch labels retained from the original analysis) in the archived dataset used to construct **Figure 5**. The original paired branch-current values and cell identifiers could not be reconstructed from the archived summary.

Material	Storage time	Individual retained ratios	*n*	Mean	Min − Max
ZnO	0 d	11.058, 5.399, 11.557, 4.909, 9.699	5	8.524	4.909–11.557
ZnO	2 d	5.857, 59.913, 13.027	3	26.266	5.857–59.913
ZnO	9 d	1.060, 0.592, 1.215, 1.119	4	0.996	0.592–1.215
AZO	0 d	0.500, 1.714, 0.267	3	0.827	0.267–1.714
AZO	3 d	523, 885, 195, 277, 139, 178, 380	7	368.143	139–885
AZO	7 d	2.840, 1.640, 1.160, 1.051	4	1.673	1.051–2.840
